# A Systematic Review of Risk Prediction Models for Esophageal Adenocarcinoma in the General Population

**DOI:** 10.1016/j.gastha.2025.100737

**Published:** 2025-06-21

**Authors:** Liyan Zhao, Binbin Chen, Jesper Lagergren, Shao-Hua Xie

**Affiliations:** 1 Department of Epidemiology and Health Statistics, School of Public Health, Fujian Medical University, Fuzhou, China; 2 Upper Gastrointestinal Surgery, Department of Molecular medicine and Surgery, Karolinska Institute, Stockholm, Sweden; 3 School of Cancer and Pharmaceutical Studies, Faculty of Life Sciences and Medicine, King’s College London, London, UK

**Keywords:** Esophageal neoplasms, Risk assessment, Critical appraisal, Precision prevention

## Abstract

**Background and Aims:**

Risk prediction models can identify individuals at high risk of esophageal adenocarcinoma. This systematic review aimed to critically appraise the available models for projecting absolute risk of esophageal adenocarcinoma in the general population.

**Methods:**

We searched Medline, Embase, and Cochrane Library databases for studies of risk prediction models for esophageal adenocarcinoma. Data were extracted from eligible studies according to the checklist for critical appraisal and data extraction for systematic reviews of prediction modelling studies. Risk of bias and applicability were assessed using the prediction model risk of bias assessment tool.

**Results:**

We identified 7 studies. Age, sex, gastroesophageal reflux disease, body mass index, and tobacco smoking were the most common predictors. The area under the receiver operating characteristic curve ranged between 0.76 and 0.88 in the derivation datasets. The models based on 2 cohort studies showed good agreement between observed and predicted risks. All studies had at least 1 domain with high risk of bias, primarily attributable to methodological shortcomings in the data analysis.

**Conclusion:**

Most risk prediction models showed good performance in identifying individuals at high risk of esophageal adenocarcinoma. Validation in external populations and cost-effectiveness evaluation are needed before these models can be applied in public health and clinical practice.

## Background

The incidence of esophageal adenocarcinoma has rapidly increased in western populations over the past 4 decades.^1^^,^^2^ Esophageal adenocarcinoma carries a poor prognosis, with an overall 5-year survival rate below 20%–30%, and tumor stage at diagnosis is the strongest prognostic factor.^3^ Early detection of tumors before symptoms occur would substantially improve the survival; the 5-year survival is >80% in patients diagnosed at stage I.^4^, ^5^, ^6^ Upper endoscopy enables detection of Barrett’s esophagus, the premalignant condition of esophageal adenocarcinoma, or esophageal adenocarcinoma at an early and curable stage.^7^^,^^8^ However, unselective endoscopic screening in the general population is not feasible due to limited health-care resources, low incidence of esophageal adenocarcinoma, and high costs and risk of complications associated with the procedure.^8^^,^^9^ Instead, selection of a limited group of individuals at high absolute risk of esophageal adenocarcinoma, who may benefit from targeted prevention and screening programs, may be a more feasible and effective strategy.

Risk prediction models that combine information on risk factors is a promising approach for selecting high-risk individuals, and such models are increasingly available for various cancer types.^10^, ^11^, ^12^, ^13^ The National Cancer Institute of United States has identified risk prediction as ‘an area of extraordinary opportunity’ early in mid-2000s.^14^ In recent years, a number of risk prediction models for projecting the absolute risk of esophageal adenocarcinoma have been developed. However, the performance and utility of these models remain to be assessed before being used in public health and clinical practice.

This systematic review aimed to critically appraise the existing risk prediction models for esophageal adenocarcinoma regarding development and validation methodologies, performance, and public health and clinical usefulness.

## Materials and Methods

This systematic review was carried out following the Preferred Reporting Items for Systematic reviews and Meta-Analyses (PRISMA) guidelines.^15^

### Literature Search

We conducted a comprehensive search in Medline, Embase, and Cochrane Library databases through February 22, 2024, for multivariable risk prediction models for projecting individuals’ absolute risk of esophageal adenocarcinoma. The detailed search strategy is presented in Tables A1-A3. Studies were included if they were original research articles developing algorithms or models for estimating the future risk of developing esophageal adenocarcinoma in individuals in the general population. Studies were excluded if they met any of the following criteria: (1) animal studies, systematic reviews, case reports, conference abstracts, validation-only studies, or methodology studies; (2) the outcomes included the precancerous lesion Barrett’s esophagus; (3) participants were not from the general population, eg, participants only including patients with gastroesophageal reflux disease or Barrett’s esophagus; and (4) studies published in languages other than English. Two authors (L.Z. and B.C.) independently screened the records to identify eligible studies. Any disagreements between the 2 reviewers were thoroughly discussed and resolved together with a senior researcher (S.H.X).

### Data Extraction

Data were systematically extracted from the eligible studies according to the checklist for critical appraisal and data extraction for systematic reviews of prediction modelling studies (CHARMS).^16^ The extracted data covered the following aspects according to CHARMS: source of data, participants, outcome to be predicted, candidate predictors, sample size, handling of missing data, model development, model performance, model evaluation, presentation of results, and interpretation of the model.

### Quality Assessment

We assessed the risk of bias and applicability of the included studies using the prediction model risk of bias assessment tool (PROBAST).^17^ The PROBAST evaluates the prediction models for the following 4 domains: selection of participants, assessment of predictors, determination of outcomes, and data analysis. For each domain, various signaling questions are answered for determining whether the risk of bias and the applicability should be graded as low, high, or unclear. If a prediction model evaluation is judged as low on all domains relating to bias and applicability, it is assigned an overall judgment of ‘low risk of bias’ or ‘low concern regarding applicability’. If an evaluation is judged as high for at least 1 domain, the model is judged as having ‘high risk of bias’ or ‘high concern regarding applicability’. If the evaluation of a model is unclear in 1 or more domains and rated as low in the remaining domains, the model is judged as of ‘unclear risk of bias’ or ‘unclear concern regarding applicability’. In the data analysis domain, the risk of bias is assessed based on key statistical considerations, including the number of participants with the outcome, handling of continuous and categorical predictors, missing data, complexities in the data, assessment of calibration and discrimination, model overfitting, and agreement of predictors’ weights in the final model. Specifically, we assessed the reasonability of the number of participants with the outcome according to the number of events per predictor variable (EPV), defined as the number of esophageal adenocarcinoma patients divided by the number of predictor variables. Models with EPVs <10 are likely to have overfitting, whereas those with EPVs ≥20 are less likely to have overfitting.^17^

## Results

### Literature Search and Included Studies

**Figure 1:**
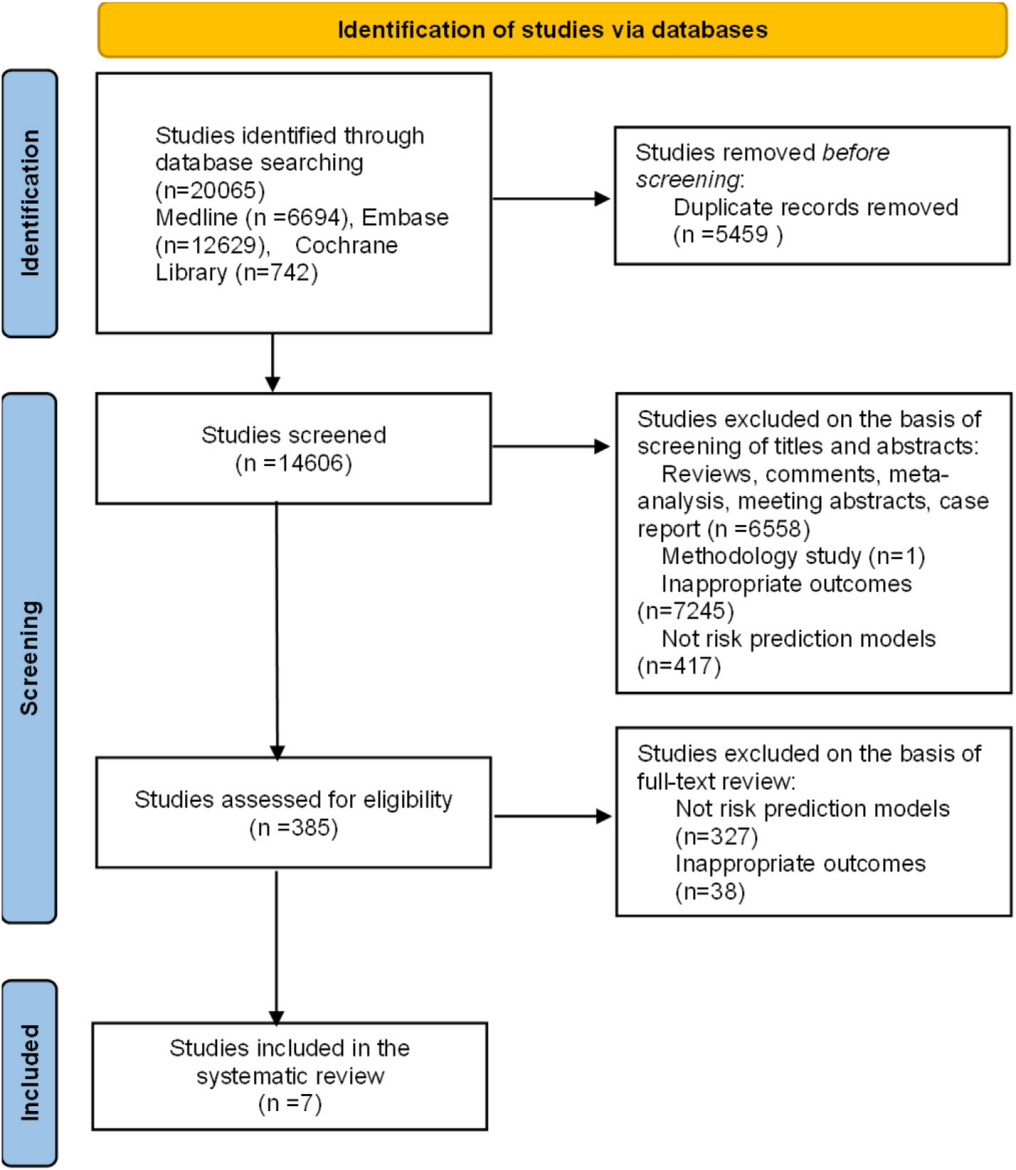
Flowchart of literature search and study selection.

A flowchart of the systematic review and study selection is presented in Figure 1. Out of 20,065 studies identified in the initial literature search, 19,680 were excluded after screening by title and abstract, leaving 385 for full-text review. Seven studies met the inclusion criteria and were included in this systematic review.^18^, ^19^, ^20^, ^21^, ^22^, ^23^, ^24^ Among these, 1 study developed 2 models based on different panels of predictors,^19^ and another developed 2 models using different algorithms (coefficient-based and point-based),^20^ allowing 9 models to be included.

The characteristics of included studies are summarized in Table 1. Four studies developed models in population-based cohorts or case-control studies in Europe (Norway, Sweden, and the United Kingdom),^19^, ^20^, ^21^, ^22^ 1 developed the model in a case-control study in Australia,^18^ 1 was based on electronic health records of hospitalized patients in the United States,^24^ and 1 used relative risk estimates from published observational studies and clinical trials.^23^

**Table 1:** Summary of Included Studies of Esophageal Adenocarcinoma

First author (year)	Country	Study design	Model type	Number of participants	Number of cases	Number of predictors	Discrimination AUC (95%CI)	Calibration methods
Thrift AP (2013)	Australia	Case-control	Gail model	1944	364	7	0.76 (0.73–0.79) (D) 0.75 (0.66–0.84) (IV)	-
Xie SH (2016)	Sweden	Case-control	Gail model	1009	189	5 (simple model) 10 (full model)	0.82 (0.78–0.85) (simple model) (D) 0.80 (0.77–0.84) (simple model) (IV) 0.84 (0.81–0.87) (full model) (D) 0.83 (0.80–0.86) (full model) (IV)	-
Xie SH (2018)	Norway	Cohort	Competing-risk regression	62,576	29	5	0.81 (0.70–0.91) (10-y risk) (D) 0.71 (0.57–0.85) (10-y risk) (IV) 0.88 (0.83–0.93) (15-y risk) (D) 0.84 (0.76–0.91) (15-y risk) (IV)	Calibration plot
Kunzmann AT (2018)	United Kingdom	Cohort	Logistic	355,034	220	5	0.80 (0.77–0.8c) (Coefficient-based) (D) 0.79 (0.77–0.8c) (Coefficient-based) (IV) 0.80 (0.77–0.82) (Points-based) (D)	Calibration plot
Kunzmann AT (2019)	United Kingdom	Cohort	Logistic	329,643	214	6	0.80 (0.77–0.83) (D)	-
Vaughan TL (2019)	NA	NA	Gail model	NA	NA	10	0.81 (0.79–0.83) (EV)	-
Iyer PG (2023)	America	Case-control	Machine learning	262,291	1539	10	0.84 (IV)	-

AUC, area under the receiver operating characteristic curve; CI, confidence interval; D, derivation; EV, external validation; IV, internal validation; NA, not applicable.

### Model Development

The included models were developed using any of the following methods: Gail model (n = 4), logistic regression (n = 3), competing-risks regression (n = 1), and machine learning (n = 1).

Candidate predictors in these models were selected based on existing literature and clinical considerations. The EPV was >20 in 6 models, 10-20 in 1 model, <10 in 1 model, and was not assessed for 1 model. Continuous variables, including age and body mass index (BMI), were converted to categorical variables in all models. Handling of missing data was reported for most models (n = 8), either by excluding participants with missing data (n = 7) or using a ‘transformer-based’ machine learning model (n = 1). More detailed methodological characteristics of the included models are presented in Table 2.

**Table 2:** Characteristics in Data Analysis for the 9 Risk Prediction Models for Esophageal Adenocarcinoma

Characteristics	Number (%)
Number of events per predictor	
<10	1 (11%)
10–20	1 (11%)
≥21	6 (67%)
Not reported	1 (11%)
Handling of continuous variables	
Transforming to categorical variables	9 (100%)
Variable selection	
Based on literature	9 (100%)
Missing data	
Excluded	7 (78%)
Transformer model	1 (11%)
Not reported	1 (11%)
Model performance	
Discrimination	9 (100%)
Calibration	2 (22%)
Validation	
Internal only	5 (56%)
External only	1 (11%)
Internal and external	1 (11%)
Not reported	2 (22%)
Internal validation	
Cross-validation	4 (67%)
Bootstrap	1 (17%)
Split-sample	1 (17%)
Model presentation	
An interactive tool	6 (67%)
Calculation formula	1 (11%)
Nomogram	2 (22%)

### Model Performance

The predictors included in the final models are presented in Table 3. The number of predictors in each model ranged between 5 and 10, and the most frequently included predictors were age, sex, smoking, BMI, and gastroesophageal reflux disease. In addition, history of diagnosis or treatment for esophageal conditions was included in 4 models, and the specific esophageal conditions for these models are provided in Table 3; family history of cancer (any type or esophageal cancer) was included in 2 models.

**Table 3:** Predictors Included in the Risk Prediction Models for Esophageal Adenocarcinoma

Models	Age	Sex	Smoking	BMI	GERD symptoms	Esophageal conditions^a^	Family history	Others
Thrift AP (2013)	√	√	√	√	√			Highest level of education, use of NSAIDs
Xie SH (simple model) (2016)	√	√	√	√	√			
Xie SH (full model) (2016)	√	√	√	√	√	√		Duration of being married or cohabiting, previous surgery for gastric or duodenal ulcer, previous surgery for esophagitis, diaphragmatic hernia or severe reflux symptoms
Xie SH (2018)	√	√	√	√	√			
Kunzmann AT (coefficient-based) (2018)	√	√	√	√		√		
Kunzmann AT (Points-based) (2018)	√	√	√	√		√		
Kunzmann AT (2019)	√	√	√	√		√		Polygenic risk scores
Vaughan TL (2019)	√	√	√	√	√		√	Physical activity, use of NSAIDs, use of statin drugs. Barrett’s screening status
Iyer PG (2023)	√	√	√	√	√		√	Race, comorbidities, medications, laboratory values.

√Indicating that the risk factor was included in the model.

BMI, body mass index; GERD, gastroesophageal reflux disease; NSAID, nonsteroidal anti-inflammatory drug.

a Esophageal conditions included: (1) for Xie SH, 2016: previously diagnosed esophagitis, diaphragmatic hernia, specific gastrointestinal operations (gastric/duodenal ulcer surgery, or operations for esophagitis/diaphragmatic hernia/severe reflux); (2) for Kunzmann AT, 2018 and Kunzmann AT, 2019: self-reported history of gastroesophageal reflux disease, Barrett's esophagus, hiatus hernia, or esophageal stricture and/or esophageal fundoplication or hiatus hernia surgery and/or antireflux medication use (none or any).

**Figure 2:**
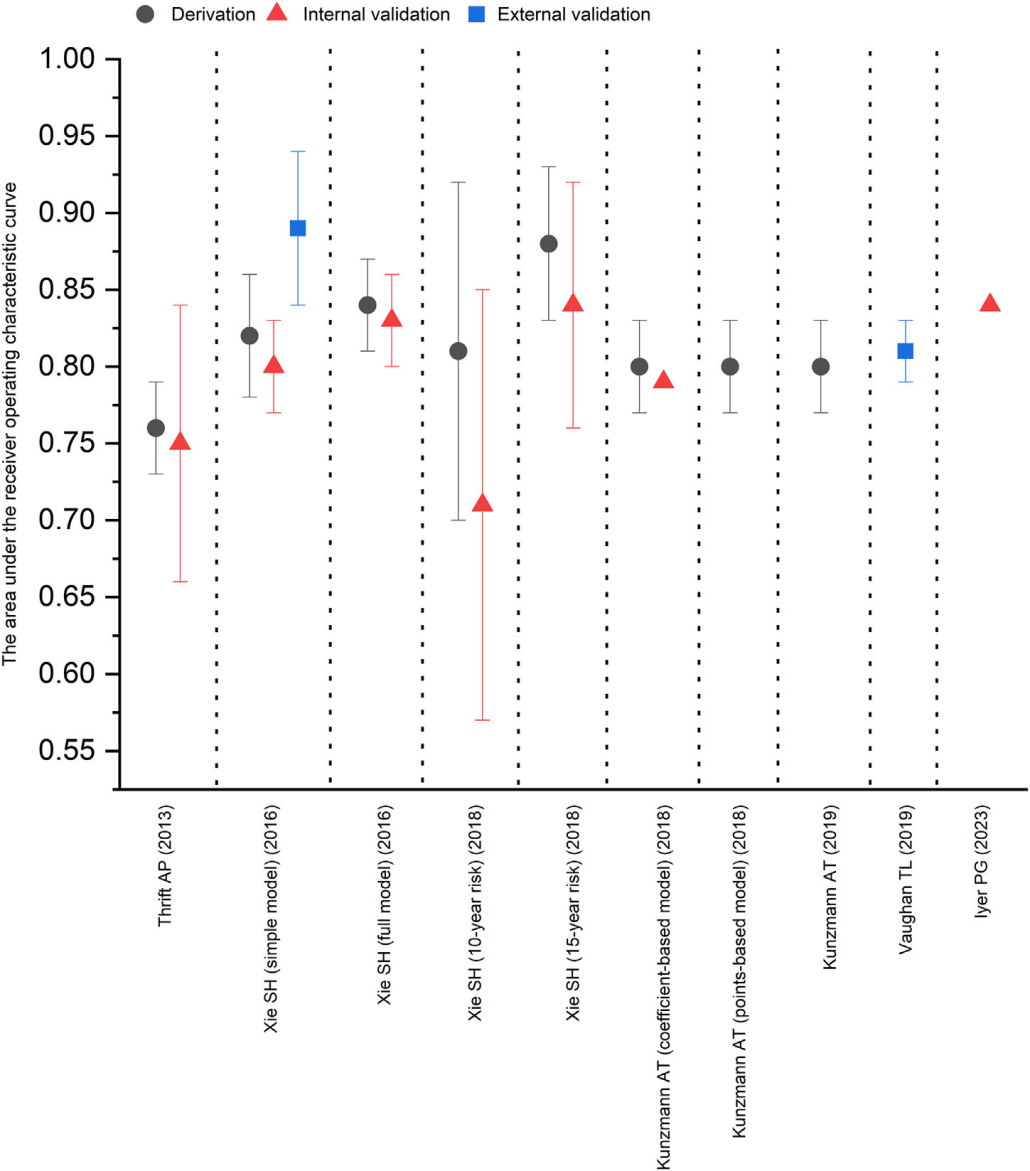
The area under the receiver operating characteristic curve of risk prediction models in derivation and validation datasets.

The discriminative ability of the models was evaluated by calculating the area under the receiver operating characteristic curve (AUC) in the derivation datasets for 7 models (range 0.76-0.88).^18^, ^19^, ^20^, ^21^, ^22^ The discriminative ability was assessed using cross-validation and bootstrap internal validation for 6 models (AUC 0.71-0.84),^18^, ^19^, ^20^^,^^22^^,^^24^ and externally validated in an independent population for 1 model (AUC 0.89).^22^ The AUC of the model based on published literature was 0.81 as assessed by using harmonized data from 6 population-based studies from the Barrett’s and Esophageal Adenocarcinoma Consortium,^23^ which provided relative risk estimates for key predictors (Figure 2). The models based on 2 population-based cohort studies (HUNT study and UK Biobank) showed good risk calibration, ie, agreement between the predicted and observed risks.^22^

### Presentation of Models

The published models were presented in 3 forms, ie, interactive tool (n = 6), calculation formula (n = 1), and nomogram (n = 2).

### Study Quality and Risk of Bias

The overall study quality and risk of bias of the included studies are summarized in Table 4 and Figure A1. Three studies exhibited a high risk of bias in the domain of participant selection, 4 studies showed a high risk of bias in the domain of predictor assessment, and all studies were rated as high risk of bias in the domain of data analysis. The detailed risk of bias assessments for each study across these domains are presented in Table A4. Two studies were rated as having high concerns regarding applicability, primarily due to issues related to the availability of information on selected predictors in the general population.

**Table 4:** Risk of Bias and Applicability of Included Studies on Esophageal Adenocarcinoma According to PROBAST

First author (year)	Risk of bias				Applicability			Overall	
	Participants	Predictors	Outcome	Analysis	Participants	Predictors	Outcome	Risk of bias	Applicability
Xie SH (2018)	+	+	+	−	+	+	+	−	+
Xie SH (2016)	−	−	+	−	+	+	+	−	+
Thrift AP (2013)	−	−	+	−	+	+	+	−	+
Kunzmann AT (2018)	+	+	+	−	+	+	+	−	+
Kunzmann AT (2019)	+	−	+	−	+	−	+	−	−
Iyer PG (2023)	−	−	+	−	+	−	+	−	−

+Indicating low risk of bias or low concern regarding applicability; −Indicating high risk of bias or high concern regarding applicability.

PROBAST, prediction model risk of bias assessment tool.

## Discussion

This systematic review identified 9 prediction models for projecting individuals’ absolute risk of esophageal adenocarcinoma in the general population. Age, sex, gastroesophageal reflux disease, BMI, and tobacco smoking were the most common predictors. The models generally showed good performance, particularly for discriminative accuracy. All included studies showed high risk of bias, primarily regarding data analysis.

Gastroesophageal reflux disease is a well-established risk factor for esophageal adenocarcinoma, and the tumor development process involves erosive esophagitis progressing to Barrett’s esophagus without dysplasia, low-grade dysplasia, high-grade dysplasia, and eventually adenocarcinoma.^6^ Upper endoscopy is often used in the diagnosis and management of reflux, particularly for identifying individuals with Barrett’s esophagus, who are included in endoscopic surveillance programs. However, it has been estimated that only approximately 7% of all esophageal adenocarcinoma patients in the United States are diagnosed through screening or surveillance.^9^ Current clinical guidelines recommend endoscopic screening in older men with long-standing reflux symptoms and additional risk factors,^25^ but this strategy has not been effective because only a part of patients with reflux symptoms have an endoscopy and 40% of esophageal adenocarcinoma patients without reflux symptoms are missed.^9^ Instead, risk prediction modelling that combines information on multiple risk factors is a promising tool to identify individuals at high risk of esophageal adenocarcinoma who may benefit from targeted primary prevention, screening, or other interventions.

This systematic review has identified 9 models for predicting individuals’ esophageal adenocarcinoma risk in the general population, which were based on various study designs and statistical methods. When assessed according to the PROBAST tool, most models showed low risk of bias in the domains of participant selection and outcome assessment, but all models showed a high risk of bias in the domain of data analysis. The major issues were inadequate handling of missing data and lack of comprehensive performance measures. The models showed good discriminative accuracy, ie, ability to discriminate between esophageal adenocarcinoma patients and participants without this cancer, as assessed by AUC and other statistics. However, assessment of risk calibration, ie, agreement between predicted and observed risks, was possible only for 3 models (based on cohort studies). This might be due to the rarity of large cohort studies with valid data on the main risk factors for esophageal adenocarcinoma. To prevent the problem of overfitting when performance of a model is assessed with the same derivation dataset, and assess the generalizability of the model, a risk prediction model needs to be validated in a separate dataset, preferably in an independent external population. However, very few of the existing risk prediction models were externally validated. Only the model, derived from a nationwide population-based case-control study in Sweden,^19^ has been validated in a fully independent population, ie, the HUNT study in Norway, showing good discriminative ability and risk calibration.^22^ A validation-only study in the United States, which was not included in this systematic review, showed that the models based on the 2 population-based cohort studies, ie, HUNT study and UK Biobank had higher accuracy than reflux symptoms for predicting the risk of esophageal adenocarcinoma.^26^ Nevertheless, it is necessary to validate the existing models in independent populations before they may be introduced in clinical practice.

The selection of predictors to be included in risk prediction models should be based on both clinical and statistical considerations. Inclusion of a limited number of relevant predictors for which information is readily available is ideal for use in clinical settings. All the existing prediction models for esophageal adenocarcinoma risk included the risk factors age, sex, gastroesophageal reflux disease, obesity, and tobacco smoking, and a simple model including these predictors only showed good performance.^22^ These predictors are all well-established risk factors for esophageal adenocarcinoma and information on these are usually readily available either in epidemiological studies or routine clinical settings. Although patients with Barrett’s esophagus have substantially elevated risk of esophageal adenocarcinoma,^6^ the majority of the identified models did not include Barrett’s esophagus as a predictor,^18^^,^^22^, ^23^, ^24^ probably due to unavailability of such information. In addition, inclusion of Barrett’s esophagus may not be realistic in real-world settings because the diagnosis is usually not available for individuals without reflux symptoms. Inclusion of additional predictors beyond the 5 main risk factors of esophageal adenocarcinoma has shown only marginal improvements in model performance, and particularly, information on genetic variants seems unlikely to increase the identification of individuals at high risk of esophageal adenocarcinoma.^21^ Previous studies have found that higher circulating levels of certain metabolic and inflammatory biomarkers, including leptin, glucose, insulin, C-reactive protein, interleukin 6, and soluble tumor necrosis factor receptor 2, are associated with an increased risk of esophageal adenocarcinoma.^27^ Thus, it would be interesting to assess whether inclusion of these biomarkers can improve the performance of prediction models in identifying individuals at high risk of esophageal adenocarcinoma. Yet, such biomarkers are not routinely tested in the general population, which limit their applicability.

There remain barriers to implementation of risk prediction models for esophageal adenocarcinoma in clinical practice. In addition to the lack of external validation, the absolute risk of esophageal adenocarcinoma among individuals with high relative risk seems not sufficiently high for considering a screening program or clinical intervention. For example, we have previously used the Lorenz curve to assessed the ‘concentration’ of esophageal adenocarcinoma patients in high-risk individuals, according to a risk prediction model developed in the HUNT cohort.^22^ It indicated that the model would capture 33% of all esophageal adenocarcinoma cases from the 5% of the population with the highest risks within 15 years, but the thresholds of the 15-year risk to include the top 5% of the population at the highest risk was as low as 191 per 100,000 persons, meaning that 523 persons needed to be surveyed to detect 1 esophageal adenocarcinoma case in 15 years. Yet, optimal thresholds of the predicted risk need to be carefully determined by taking into account the incidence (absolute risk) of esophageal adenocarcinoma in a given population, potential benefits, and harms for the individuals, as well as health-care resources, which depends on the specific screening techniques or interventions to be implemented. Cost-effectiveness analysis is necessary to evaluate the feasibility of any prevention programs based on individualized risk assessment.

Strengths of this study include the updated comprehensive literature search and critical appraisal of the models using the CHARMS and PROBAST tools. Compared with earlier systematic reviews,^28^^,^^29^ this study included 2 recent models using novel methodologies. One of these used robust data largely derived from meta-analyses of pooled individual data, which increased the generalizability of the model.^23^ The other machine learning model based on an electronic health record database considered the temporal aspects of the clinical features and also allowed for missing features for a patient, which is relevant if the model is implemented clinically.^24^ There are also several limitations of this systematic review. First, it did not include models predicting the risk of Barrett’s esophagus. However, this condition is considered a different outcome and only a small proportion (<0.2% annually) of patients with Barrett’s esophagus develop esophageal adenocarcinoma.^30^ Second, the search only included studies published in English. But this should be a minor issue because studies examining models to predict esophageal adenocarcinoma are less likely to be published in other languages. Third, meta-analysis was not conducted for the measures of model performance because of the substantial heterogeneity across the models, including the differences in incidence across populations and varying panels of predictions with different definitions and assessment.

## Conclusion

In summary, this systematic review critically appraised the existing risk prediction models for projecting individuals’ risk of esophageal adenocarcinoma in the general population. It examined the methods for development and validation, predictive performance, risk of bias, and the applicability of the models. These models showed good performance and promising potential in selecting individuals at high risk of esophageal adenocarcinoma for targeted prevention and screening compared with the failed strategies relying on reflux symptoms only. However, these models require further validation in external populations and cost-effectiveness evaluation before they may be applied in public health and clinical practice.
