# Supplementary material for: A Systematic Review of Risk Prediction Models for Esophageal Adenocarcinoma in the General Population
Source: Gastro Hep Adv. 2025 Jun 21;4(10):100737. doi: 10.1016/j.gastha.2025.100737 (PMC12409981; doi:10.1016/j.gastha.2025.100737)
Supplement: Supplementary materials [file mmc1.docx]

Supplementary materials

**Supplementary Table S1.** Search strategies in Medline

**Supplementary Table S2.** Search strategies in Embase

**Supplementary Table S3.** Search strategies in Cochrane Library

**Supplementary Table S4.** Risk of bias of included studies using the prediction study risk of bias assessment tool (PROBAST)

**Supplementary Figure S1.** Risk of bias (A) and applicability (B) of included models according to the prediction model risk of bias assessment tool (PROBAST)

| **Supplementary Table S1.** Search strategies in Medline | |
| --- | --- |
| **Step** | **Key words** |
| #1 | "oesophagus"[Title/Abstract] OR "esophagus"[Title/Abstract] OR "esophageal"[Title/Abstract] OR "oesophageal"[Title/Abstract] OR "upper digestive"[Title/Abstract] OR "upper aerodigestive"[Title/Abstract] OR "upper gastrointestinal"[Title/Abstract] |
| #2 | "cancer"[Title/Abstract] OR "carcinoma"[Title/Abstract] OR "malignan*"[Title/Abstract] OR "neoplasm"[Title/Abstract] OR "tumour"[Title/Abstract] OR "tumor"[Title/Abstract] OR "adenocarcinoma"[Title/Abstract] |
| #3 | #1 AND #2 |
| #4 | "Esophageal Neoplasms"[MeSH Terms] |
| #5 | #3 OR #4 |
| #6 | "risk"[Title/Abstract] OR "predict*"[Title/Abstract] OR "prognos*"[Title/Abstract] |
| #7 | "model"[Title/Abstract] OR "equation"[Title/Abstract] OR "calculat*"[Title/Abstract] OR "scor*"[Title/Abstract] OR "algorithm"[Title/Abstract] |
| #8 | #6 AND #7 |
| #9 | #5 AND #8 |

| **Supplementary Table S2.** Search strategies in Embase | |
| --- | --- |
| **Step** | **Key words** |
| #1 | (esophagus):ti,ab OR (oesophagus):ti,ab OR (esophageal):ti,ab OR (oesophageal):ti,ab OR (upper digestive):ti,ab OR (upper aerodigestive):ti,ab OR (upper gastrointestinal):ti,ab |
| #2 | (cancer):ti,ab OR (carcinoma):ti,ab OR (malignan*):ti,ab OR (neoplasm):ti,ab OR (tumour):ti,ab OR (tumor):ti,ab OR (adenocarcinoma):ti,ab |
| #3 | #1 AND #2 |
| #4 | Esophageal Neoplasms/exp |
| #5 | #3 OR #4 |
| #6 | (risk):ti,ab OR (predict*):ti,ab OR (prognos*):ti,ab |
| #7 | (model):ti,ab OR (equation):ti,ab OR (calculat*):ti,ab OR (scor*):ti,ab OR (algorithm):ti,ab |
| #8 | #6 AND #7 |
| #9 | #5 AND #8 |

| **Supplementary Table S3.** Search strategies in Cochrane Library | |
| --- | --- |
| **Step** | **Key words** |
| #1 | (esophagus):ti,ab OR (oesophagus):ti,ab OR (esophageal):ti,ab OR (oesophageal):ti,ab OR (upper digestive):ti,ab OR (upper aerodigestive):ti,ab OR (upper gastrointestinal):ti,ab |
| #2 | (cancer):ti,ab OR (carcinoma):ti,ab OR (malignan*):ti,ab OR (neoplasm):ti,ab OR (tumour):ti,ab OR (tumor):ti,ab OR (adenocarcinoma):ti,ab |
| #3 | #1 AND #2 |
| #4 | MeSH descriptor:[Esophageal Neoplasms] |
| #5 | #3 OR #4 |
| #6 | (risk):ti,ab OR (predict*):ti,ab OR (prognos*):ti,ab |
| #7 | (model):ti,ab OR (equation):ti,ab OR (calculat*):ti,ab OR (scor*):ti,ab OR (algorithm):ti,ab |
| #8 | #6 AND #7 |
| #9 | #5 AND #8 |

| **Supplementary Table S4.**  Risk of bias of included studies using the prediction study risk of bias assessment tool (PROBAST) | | | | | | | | | | | | | | | | | | | | |  |
| --- | --- | --- | --- | --- | --- | --- | --- | --- | --- | --- | --- | --- | --- | --- | --- | --- | --- | --- | --- | --- | --- |
| **First author (year)** | **Participants** | | **Predictors** | | | **Outcome** | | | | | | **Analysis** | | | | | | | | | |
|  | 1.1 | 1.2 | 2.1 | 2.2 | 2.3 | 3.1 | 3.2 | 3.3 | 3.4 | 3.5 | 3.6 | 4.1 | 4.2 | 4.3 | 4.4 | 4.5 | 4.6 | 4.7 | 4.8 | 4.9 | |
| Xie SH (2018) | Y | Y | Y | Y | Y | Y | Y | Y | Y | Y | Y | N | Y | Y | N | Y | Y | Y | Y | Y | |
| Xie SH (2016) | N | Y | Y | N | Y | Y | Y | Y | Y | Y | Y | Y | Y | Y | N | Y | Y | N | Y | Y | |
| Thrift AP (2013) | N | N | Y | N | Y | Y | Y | Y | Y | Y | Y | Y | Y | Y | N | Y | Y | N | Y | Y | |
| Kunzmann AT (2018) | Y | Y | Y | Y | Y | Y | Y | Y | Y | Y | Y | Y | Y | Y | N | Y | N | Y | Y | Y | |
| Kunzmann AT (2019) | Y | Y | Y | Y | N | Y | Y | Y | Y | Y | Y | Y | Y | Y | N | Y | N | N | N | Y | |
| Iyer PG (2023) | N | N | Y | N | N | Y | Y | Y | Y | Y | Y | Y | Y | Y | Y | Y | Y | N | N | Y | |
| Abbreviation: Y=Yes, N=No, NI=No information  Q1.1: Were appropriate data sources used, e.g., cohort, RCT, or nested case–control study data?  Q1.2: Were all inclusions and exclusions of participants appropriate?  Q2.1: Were predictors defined and assessed in a similar way for all participants?  Q2.2: Were predictor assessments made without knowledge of outcome data?  Q2.3: Are all predictors available at the time the model is intended to be used?  Q3.1: Was the outcome determined appropriately?  Q3.2: Was a prespecified or standard outcome definition used?  Q3.3: Were predictors excluded from the outcome definition?  Q3.4: Was the outcome defined and determined in a similar way for all participants?  Q3.5: Was the outcome determined without knowledge of predictor information?  Q3.6: Was the time interval between predictor assessment and outcome determination appropriate?  Q4.1: Were there a reasonable number of participants with the outcome?  Q4.2: Were continuous and categorical predictors handled appropriately?  Q4.3: Were all enrolled participants included in the analysis?  Q4.4: Were participants with missing data handled appropriately?  Q4.5: Was selection of predictors based on univariable analysis avoided?  Q4.6: Were complexities in the data (e.g., censoring, competing risks, sampling of control participants) accounted for appropriately?  Q4.7: Were relevant model performance measures evaluated appropriately?  Q4.8: Were model overfitting and optimism in model performance accounted for?  Q4.9: Do predictors and their assigned weights in the final model correspond to the results from the reported multivariable analysis? | | | | | | | | | | | | | | | | | | | | | |


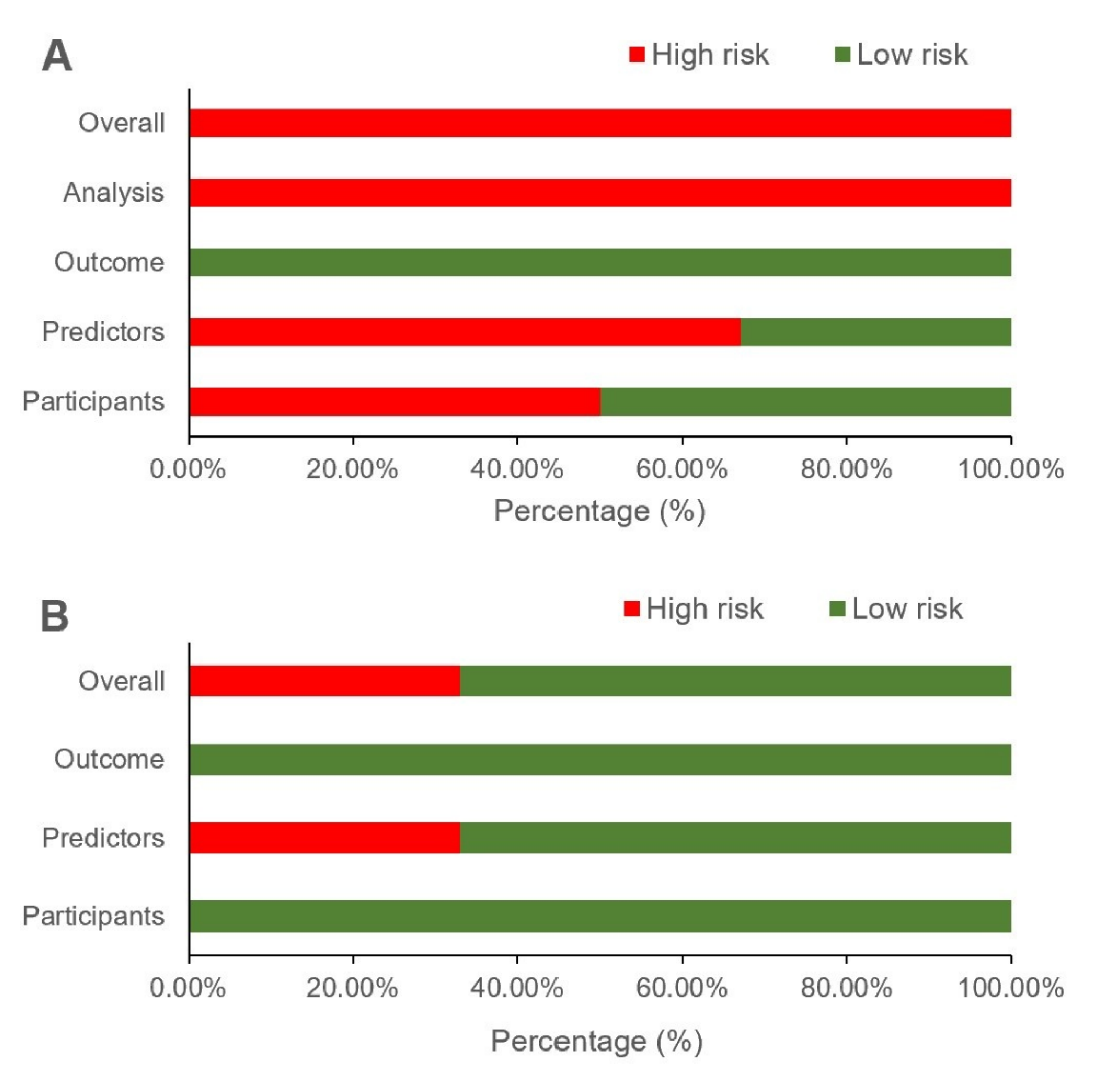


**Supplementary Figure S1.** Risk of bias (A) and applicability (B) of included models according to the prediction model risk of bias assessment tool (PROBAST).
